# Kolmogorov–Arnold and Long Short-Term Memory Convolutional Network Models for Supervised Quality Recognition of Photoplethysmogram Signals

**DOI:** 10.3390/e27040326

**Published:** 2025-03-21

**Authors:** Aneeqa Mehrab, Michela Lapenna, Ferdinando Zanchetta, Angelica Simonetti, Giovanni Faglioni, Andrea Malagoli, Rita Fioresi

**Affiliations:** 1 Department of Mathematics, University of Ferrara, Via Ariosto 35, 44122 Ferrara, Italy; aneeqa.mehrab@unife.it; 2 FaBiT, University of Bologna, Via San Donato 15, 40127 Bologna, Italy; michela.lapenna4@unibo.it (M.L.); ferdinando.zanchett2@unibo.it (F.Z.); 3 Department of Economics, University of Chieti-Pescara “G. d’Annunzio”, Viale Pindaro 43, 65127 Pescara, Italy; angelica.simonetti@gmail.com; 4 Nabla2, Viale Monchio 116, 41124 Modena, Italy; giova@nabla2.it; 5 VST, Via Carlo Zucchi, 21d, 41123 Modena, Italy; andrea.malagoli@unimore.it

**Keywords:** PPG signals, convolutional neural networks, long short-term memory, Kolmogorov–Arnold networks

## Abstract

Photoplethysmogram (PPG) signals recover key physiological parameters as pulse, oximetry, and ECG. In this paper, we first employ a hybrid architecture combining the Convolutional Neural Network (CNN) and Long Short-Term Memory (LSTM) for the analysis of PPG signals to enable an automated quality recognition. Then, we compare its performance to a simpler CNN architecture enriched with Kolmogorov–Arnold Network (KAN) layers. Our results suggest that the usage of KAN layers is effective at reducing the number of parameters, while also enhancing the performance of CNNs when equipped with standard Multi-Layer Perceptron (MLP) layers.

## 1. Introduction

**Figure 1:**
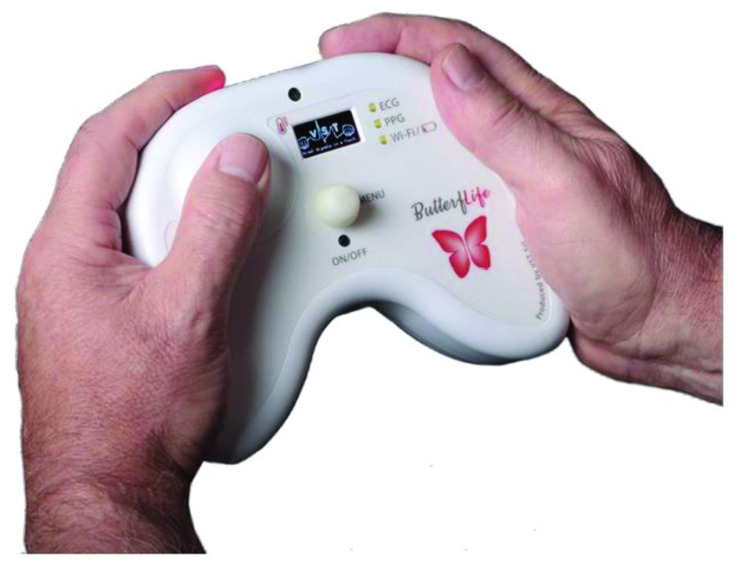
The ButterfLife device, which captures PPG signals at 512 Hz for any time interval.

PPG is a popular, contact, and noninvasive optical measurement technique for recording variations in blood volume in the microvascular layer of the tissues [1]. From PPG signals, we can infer physiological parameters, including pulse rate, blood oxygen levels, and blood pressure. Hence, PPG is an essential signal for wearable health devices and clinical monitoring systems, for an effective and non-invasive health screening of patient status for heart-related and vascular diseases [2]. Nonetheless, PPG signals are prone to interferential noise and may be easily distorted based on motion artifacts or environmental conditions [3,4]. It might be difficult or even impossible to extract information from noisy or highly distorted signals, or the information we obtain from them might be unreliable or deceiving. This motivates the search for efficient algorithms to distinguish low-quality signals from high-quality ones and use only the latter for medical applications. The aim of this work is to develop a supervised deep learning approach based on CNN (the Convolutional Neural Network) and enhanced with LSTM (Long Short-Term Memory) or KAN (the Kolmogorov–Arnold Network) to detect anomalies in PPG timeseries data obtained via hand-held devices as in Figure 1.

CNNs are popular for image recognition and to deal with grid-like data, while LSTMs perform well with time series data [5]: the combination of these architectures can hence provide us with a modern approach to the problem of anomaly detection in PPG time series data, as already evidenced in [6,7,8], tackling similar questions to the one addressed here.

The organization of our paper is as follows. Section 2 describes the background in the literature and relevance of the question of anomaly identification in PPG signals, emphasizing the challenges posed by noise and the distortion of the signal. Section 3 details the materials and methods used in this work. In Section 3.1, we examine the preprocessing pipeline, which includes downsampling, noise reduction with the moving average and Butterworth filters, and finally Min-Max scaling to improve signal quality for the analysis described later on. Next, in Section 3.2 we examine an CNN-LSTM hybrid architecture for our model, with a focus on its capacity to capture space and time correlations. Contributions from the MLP and KAN designs are then discussed, including implementation details and hyperparameter tuning for our models. Section 3.2 also describes the dataset splitting approach, which ensures impartial training, validation, and testing. Section 4 presents our results and compares the performances of our three models: CNN-LSTM, MLP, and KAN. We compare them on criteria such as accuracy, precision, recall, F1-score, and AUC, providing insights into their strengths and limitations. Finally, Section 5 concludes our work by reviewing the findings, discussing their implications, and suggesting future research avenues for developing wearable health solutions based on PPG technology.

## 2. Related Work

The research on PPG signals has intensified over the past few years, especially with respect to cardio vascular assessment [9]. This field, now focusing more on non-invasive portable devices [10], is attracting more and more attention, especially in relation to machine learning and, more specifically, the deep learning analysis of PPG signals (see the survey [11]). In particular, several studies have addressed the challenging question of improving signal quality, detecting abnormalities, and filtering out noise (see [12] for a comprehensive survey).

However, the existing solutions, as detailed in [12], can be limiting in terms of their applicability to different datasets or when dealing with time-varying and location-related structures inherent in PPG signals coming, for example, from proprietary patented devices. Indeed, it is difficult to locate datasets with reliable labeling on the PPG signal, thus preventing meaningful comparisons between our method and others. Furthermore, there is an ongoing debate regarding the best approaches for characterizing the quality of signals; distortions are currently treated with with different hypotheses proposed for both simpler statistical analyses and more sophisticated deep learning techniques [13].

In our work, we compare our hybrid CNN-LSTM system to two other simpler models, combining CNN architectures with either Multi-Layer Perceptrons (MLPs) [14,15] or the recent Kolmogorov–Arnold Networks (KANs) [16]. This comparison has the aim to explain and understand the complexity needed by a model architecture to achieve good levels of anomaly detection. We hope that our work will stimulate the discussion and that more methods tackling this same key question regarding the quality of the signal will be developed and tested on new benchmarked datasets, not yet openly available at the moment.

## 3. Materials and Methods

In this work, we propose a novel approach including a variety of deep learning algorithms [17] and extensive preprocessing of the PPG signals for the identification of low-quality signals (anomalies), provided by the company VST. PPG signals themselves contain significant noise and are easily distorted by movement, electric and light interference, or inter and intra-individual differences. Therefore, preprocessing data and developing proper models to tackle these issues are both essential for anomaly detection. To assess our approach, we used real-world PPG signal data obtained from VST and recorded using the ButterfLife device [18], which captures PPG signals at 512 Hz for any time interval. The data recording, using the ButterfLife device, was carried out under the supervision of a medical expert and a VST-trained employee. The technical details of the ButterfLife device and its importance can be found in [18]. For data analysis, we divided the signals into 5-second segments, and the labeling was performed accordingly. In Figure 1, we show the ButterfLife device (courtesy of VST). More specifically, the ButterfLife device provides a reliable, simple, and fast simultaneous touch measurement of the five main parameters recommended by the WHO (hearth rate, respiratory rate, blood pressure, body temperature, and oxygen saturation), one-lead PPG, and ECG curve. This device allows patients to record these data without the supervision of a medical expert and allows them to share the recorded data with a doctor via a proprietary web platform. To our knowledge, this device is the only one provided with this tecnology, thus making the analysis of its signal of paramount importance [18].

The present workflow starts with cleaning the raw PPG signals to enhance the signal quality while retaining representative features. The processed signals are then split into training, validation, and test datasets to make sure that there is no information leakage between them. After this, different deep learning models are trained on the training set and validated on the validation set, the first of which combines the CNN [19,20] and LSTM [21] layers, while the other two combine the CNN with the MLP [14,15] and KAN [16] layers, respectively. The correct hyperparameters for each model are sought in a predictive way by means of a grid search. Finally, the best models of each type are deployed on the test set for the final evaluation.

### 3.1. Data Preprocessing

**Figure 2:**
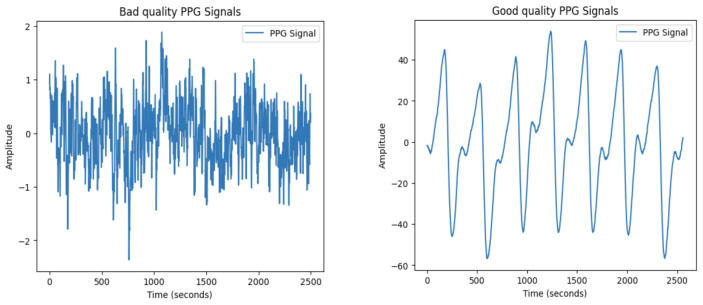
PPG signal before preprocessing (bad quality signal having label 0 vs. good-quality signal having label 1, see Section 3.2).

**Figure 3:**
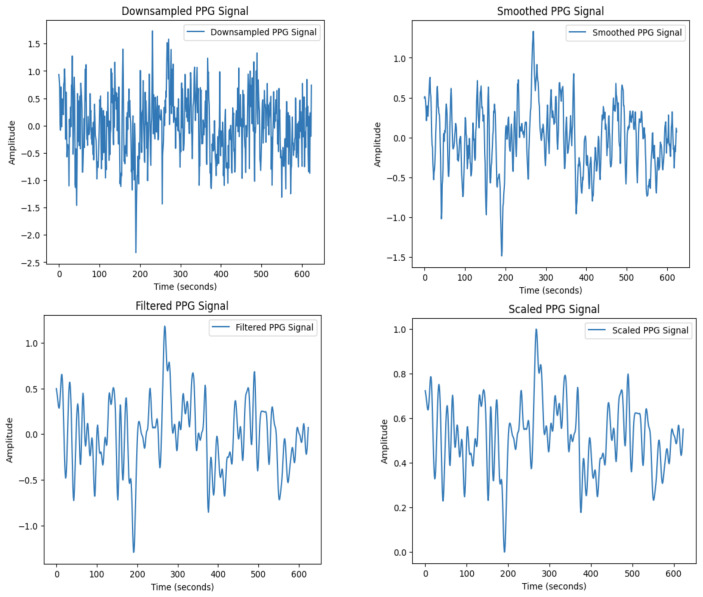
PPG sample after the four preprocessing steps are applied in sequence.

The PPG dataset consists of 5111 one-dimensional time series, each corresponding to a 5-second segment as previously mentioned. The values within each time series represent the amplitude of the PPG signal over time, measuring the variation in blood volume within the microvascular layer of the tissues. In order to optimize the analysis and model training, the preprocessing of PPG signals is crucial. Due to their sensitivity to noise and distortions from motion artifacts, and other environmental factors, PPG signals need to be preprocessed to retain important features while suppressing unwanted noise. An example of a PPG sample before preprocessing is given in Figure 2. The preprocessing steps used in this study are designed to address these challenges and are outlined in detail below (see Figure 3 for a visualization of the initial PPG sample after applying each preprocessing step).

**1. Downsampling.** Downsampling reduces the sampling frequency, and care is taken in order to preserve the important characteristics of the signal features, including the time of peaks and troughs of the heart rate. Also, before downsampling, we apply anti-aliasing, hence ensuring that the high frequencies do not affect the rest of the samples in the reduced sample. This preprocessing step limits data redundancy and its dimension, therefore making the dataset manageable for subsequent preprocessing and analysis without loosing key pieces of data.

**2. Smoothing via Moving Average Filter.** Noise often interferes with the short-term details of the PPG waveform, and while preserving the shape of the waveform, we employ a moving average filter to reduce it. This filtering step replaces a specific point amplitude in the time series with the average of the present amplitude and its neighboring points amplitude. A key parameter for this preprocessing step is the choice of the window size for the average, so that the frame’s ability to capture a signal’s frequency is not reduced. Small window sizes could not filter noise effectively enough, while large window sizes could prevent the detection of peaks and troughs in PPG signals. The window size was adjusted experimentally in order to preserve signal features and the integrity of the following signal analysis.

**3. Butterworth Low-Pass Filter.** To further address noise, especially high-frequency components introduced by motion artifacts, a Butterworth low-pass filter is applied [18].

The Butterworth filter was chosen for its flat response in the passband region where the desired signal frequencies are located in order to minimize the distortion of the frequencies. The cutoff frequency is situated at 50 Hz to capture and suppress the frequency range containing noise while preserving the lower frequencies useful in PPG. This filter reduces the high-frequency noise, thus improving the visualization of PPG waveforms, which downstream models can distinguish from real signal patterns. This step also prevents the ratio from being contaminated by distortions that can degrade the performance of machine learning algorithms.

**4. Min-Max Scaling.** Neural networks are very receptive to changes in the scale of measurements of the input parameters. To make each signal contribute equally, we normalize the range of the signal with Min-Max scaling, eliminating the effect of higher value signals dominating over the others. This step normalizes the amplitude of all signals to a range of 0 to 1. Min-Max scaling is important in the preprocessing stage because it helps to enhance the convergence properties of the neural network and prevents the risk to bias learning.

Through the above preprocessing steps, the PPG samples are normalized, cleaned, and equitably scaled. This is important in order to make sure that the machine learning models can properly learn from the data and generalize. The preprocessing pipeline establishes a good background for the following modeling and analysis stages.

### 3.2. Model Design and Training

Detecting anomalies in PPG signals requires a robust approach to capture both spatial and time dependencies. To achieve this, we develop three different models: a hybrid CNN and LSTM, a CNN model combined with classical MLP, and finally another CNN model combined with KAN. We train our models by dividing our labeled dataset into three subsets with a fixed split ensuring reproducibility: training (40%), validation (30%), and test (30%). Stratified splitting ensures uniform class distribution across subsets to avoid bias and maintain balance among signal quality classes corresponding to the three labels: 1 for good-quality signals with little interference and clear trendlines; 2 for fair quality signals, still usable albeit a moderate noise; and 3 for poor quality signals with a lot of noise, thereby inappropriate for analysis.

For the following analysis, the signals in class 2 and 3 are combined into a single fair/poor category and class 1 constitutes the good category. With this simplified binary classification, it is possible to create a model caable of separating usable PPG signals from those requiring either removal or correction.

All models are trained for a maximum of 200 epochs with an early stopping criterion (patience = 50 epochs) to prevent overfitting. Model weights from the epoch with the lowest validation loss are saved for testing. The Adam optimizer is chosen for its adaptive learning rate and computational efficiency. Binary cross-entropy loss is used for the binary classification task, following the formula:

$$L_{\mathrm{BCE}}=-\frac{1}{N}\sum_{i=1}^{N}\left[y_{i}\log\left(p_{i}\right)+\left(1-y_{i}\right)\log\left(1-p_{i}\right)\right]$$

where *N* is the number of samples, $y_{i}$ is the actual binary label (0 or 1) of the *i*-th sample, and $p_{i}$ is the predicted probability of the *i*-th sample being in class 1.

**Figure 4:**
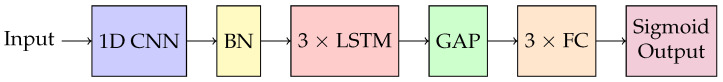
Illustration of the CNN-LSTM architecture. BN stands for batch normalization, GAP for global average pooling, and FC for fully connected layer.

**1. CNN-LSTM Architecture.** The CNN-LSTM model in Figure 4 is designed with the following components, each optimized for the task at hand:

- **The Convolutional Layer:** We first apply 1D convolutional filters to detect spatial patterns such as waveform shapes. We search for the optimal kernel size to evaluate its impact on feature extraction.
- **Batch Normalization:** We stabilize training by normalizing layer outputs, improving convergence speed and mitigating vanishing or exploding gradients.
- **The LSTM Layers:** The LSTM layer captures temporal dependencies, such as pulse timing and rhythms.
- **Global Average Pooling:** The global average pooling aggregates temporal information into a single value per feature map, reducing the sequence length.
- **Feed-Forward Layers:** Fully connected layers gradually reduce features to a single output, with a sigmoid activation function producing the final binary classification (good vs. poor/fair quality).

A grid search is performed to identify the optimal configuration of the CNN kernel size and the hidden dimension of the LSTM layer. We experiment with kernel sizes of (5, 10, and 15) and LSTM hidden dimensions of (28, 56, and 112). The selection is based on maximizing the Area Under the Curve (AUC) score on the validation set. The best-performing hyperparameter combination among all the hyperparameters we tested included a kernel size value of 15 while using an LSTM hidden dimension of 56 coupled with a feed-forward layer configuration of [112, 64].

The CNN-LSTM architecture effectively integrates spatial and temporal feature extraction capabilities. Through careful hyperparameter tuning and layer design, it achieves robust performance in anomaly detection, demonstrating its potential for biomedical signal processing applications.

**2. MLP and KAN Architectures.** MLPs, or fully connected feed-forward neural networks, are foundational in modern deep learning [14,15]. MLPs are effective at capturing complex patterns and non-linear relationships in time series data, making them valuable for classification and forecasting tasks.

As an alternative, KANs have recently been introduced [16]. Unlike MLPs, which rely on fixed activation functions, KANs feature learnable activation functions on their edges (weight connections). This unique characteristic increases flexibility and interpretability while often reducing computational complexity. Despite these advantages, KANs can suffer from slow training speeds due to their more intricate representation.

While MLPs are grounded in the universal approximation theorem [22], KANs utilize the Kolmogorov–Arnold representation theorem [23]. This theorem allows any multivariate continuous function on a bounded domain to be expressed as a composition of univariate functions and summations. KANs break high-dimensional functions into smaller, manageable components, enabling efficient learning of complex patterns.

KANs use learnable 1D functions parametrized as linear combinations of B-splines [24,25]. B-splines provide precise local adjustments and the ability to adapt resolution dynamically, making KANs well-suited for feature optimization. Their hyperparameters include: the number of basis functions constructing the activation function (*width*); the interval and granularity of the activation function (*grid*), with a finer grid capturing more detail but risking overfitting; and the smoothness of the B-spline curve (*order*), with higher orders producing smoother functions.

In an MLP, a single layer maps the input vector $x_{l}$ to the output $x_{l+1}$ as follows:

$$x_{l+1}=\sigma \left(W_{l}x_{l}+b_{l}\right)$$

where $W_{l}$ is the weight matrix of the layer *l*, $b_{l}$ is the bias term, and σ is the fixed activation function (e.g., sigmoid). When we stack multiple layers, a deep MLP with *L* layers can be written as:

$$\mathrm{MLP}\left(x\right)=\sigma^{\left(L\right)}\left(W^{\left(L\right)}\sigma^{\left(L-1\right)}\left(W^{\left(L-1\right)}\cdots \sigma^{\left(1\right)}\left(W^{\left(1\right)}x+b^{\left(1\right)}\right)\cdots +b^{\left(L-1\right)}\right)+b^{\left(L\right)}\right)$$

A single layer in a KAN transforms the input vector $x_{l}$ into the output $x_{l+1}$ using a matrix of learnable functions $\Phi_{l}$:

$$x_{l+1}=\Phi_{l}\left(x_{l}\right)$$

Expanding for each neuron:

$$x_{l+1,j}=\sum_{i=1}^{n_{l}}\phi_{l,j,i}\left(x_{l,i}\right),\;j=1,\ldots ,n_{l+1}$$

where $\phi_{l,j,i}\left(\cdot \right)$ is the learnable activation function, e.g., a unitary function parametrized with a B-spline curve. When we stack multiple layers, the KAN architecture computes:

$$\mathrm{KAN}\left(x\right)=\left(\Phi_{L-1}\circ \Phi_{L-2}\circ \cdots \circ \Phi_{1}\circ \Phi_{0}\right)x$$

**Figure 5:**
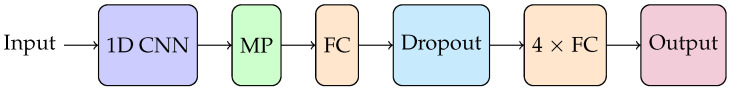
Illustration of the CNN-MLP architecture. MP stands for max pooling and FC for fully connected layer.

**Figure 6:**
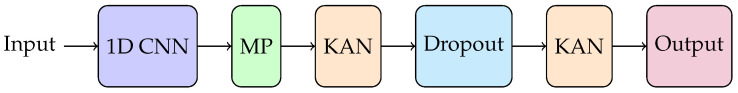
Illustration of the CNN-KAN architecture. MP stands for max pooling.

For the implementation of the CNN-MLP and CNN-KAN architectures, see Figure 5 and Figure 6. Their design includes:

- **The Convolutional Layer:** A 1D CNN expands the number of channels to a small hidden dimension while reducing input sequence length for subsequent layers.
- **Max Pooling:** Max pooling is applied to further reduce the sequence length. Its output is then averaged across channels before being fed it into the fully connected layers.
- **The Feed-Forward Layers:** The output is processed through multiple fully connected layers, gradually reducing the sequence to one dimension. A sigmoid activation function is used for the final classification in MLPs, while no a priori activation function is needed by KANs.

For the CNN-MLP architecture, the hidden dimensions of the first four feed-forward layers are tested with configurations of (140, 120, 90, and 45), (120, 100, 80, and 4), and (100, 50, 25, and 10). For KANs, the grid [2, 3, 5], order [2, 3, 5], and width [100, 80, 50] are explored. The selection is based on maximizing the AUC score on the validation set. Grid search identifies the following optimal settings: fully connected layers with hidden dimensions [100, 50, 25, and 10] for the MLP, a grid of 3, an order of 5, and a width of 80 for the KAN.

The CNN-KAN architecture demonstrates superior performance with respect to the CNN-MLP model. However, the KANs’ slower training speed remains a drawback, indicating the need for further optimization in training algorithms.

## 4. Results

Multiple evaluation metrics are used to assess the performance of the models, as listed below:

- **Accuracy:** Calculates the general percentage of correct forecasts.
  $$\mathrm{Accuracy}=\frac{TP+TN}{TP+TN+FP+FN}$$
  where TP is true positives, TN is true negatives, FP is false positives, and FN is false negatives.
- **Precision:** Computes the ratio of genuine positives in relation to all predicted positives.
  $$\mathrm{Precision}=\frac{TP}{TP+FP}$$
- **Recall:** Measures the percentage of real positives over all true positives.
  $$\mathrm{Recall}=\frac{TP}{TP+FN}$$
- **F1-Score:** The harmonic mean of precision and recall.
  $$F1\text{-}\mathrm{Score}=2\times \frac{\mathrm{Precision}\times \mathrm{Recall}}{\mathrm{Precision}+\mathrm{Recall}}$$
- **AUC:** Evaluates the capability of the model to distinguish between the two classes across different thresholds.
  $$\mathrm{AUC}=\int_{0}^{1}\mathrm{TPR}\left(t\right)\;d\left(\mathrm{FPR}\left(t\right)\right)$$
  where TPR(t) is the true positive rate and FPR(t) is the false positive rate at a given threshold *t*.

Table 1 reports the evaluation metrics for the three models with the best hyperparameter configuration when evaluated on the test set.

In Table 2, we compare the three architectures in terms of their number of trainable parameters.

The results demonstrate that the models achieve good performance across all metrics, particularly in terms of AUC. The CNN-LSTM architecture outperforms the simpler feed-forward models, excelling in distinguishing between poor/fair and good-quality PPG signals. However, this performance comes at the cost of a significantly higher number of trainable parameters. Conversely, the MLP and KAN architectures achieve respectable performance with drastically fewer parameters. Notably, the KAN model, despite having only two layers, outperforms the five-layer MLP model. We stress, however, that KAN’s number of trainable parameters is greater with respect to the MLP model.

## 5. Discussion

The evaluation results verify the effectiveness of the proposed models for distinguishing good-quality signals from low-quality ones. The high AUC values observed for all models substantiate their good capabilities of signal quality classification between both classes. CNN-LSTM is found to perform better than the other models, a result primarily due to combining the CNN ability to capture spatial features and the LSTM one to capture temporal characteristics. This implies that convolutional and recurrent layered learning is appropriate for time-series data with spatial and temporal dimensions.

The simpler CNN-MLP and CNN-KAN models give slightly less satisfactory results with respect to CNN-LSTM but with a lower number of training parameters. Specifically, the CNN-KAN model, while being less complicated than the above-mentioned CNN-LSTM architecture, yields better results in comparison with the CNN-MLP model. In particular, the CNN-KAN performs only slightly below the CNN-LSTM model, the best-performing model, though CNN-KAN employs only one third of the trainable parameter with respect to CNN-LSTM; hence, it represents a step towards sustainability goals.

It should also be mentioned that there are a lot of preprocessing steps to make the data clean in order to feed them to models. Also, our findings also highlight some drawbacks, including the fact that the sub-optimal quality of manual labeling can introduce bias/inconsistencies within the training data.

## 6. Conclusions

This research proves the effectiveness of employing a CNN-LSTM to determine the quality and detect anomalies in PPG signals. The proposed CNN-LSTM model outperforms simpler CNN models by combining CNN’s ability to capture spatial relationships with LSTM’s ability to capture temporal ones. Combining CNN with simpler feed-forward networks, such as MLP and KAN, also yields good results and is then recommended when computational resources are limited.

We also show how critical preprocessing is used to enhance the data quality and then to enable successful anomaly detection, while hyperparameter tuning through grid search is crucial to enhance each model performance.

Future work can aim to further refine quality assurance but should come together with a reliable labeling of data, which we plan to implement in a further study.

## Figures and Tables

**Table 1:** Evaluation metrics on the test set for the three architectures. For the readers, convenience we report them all in percentage format.

Metric	CNN-LSTM	MLP	KAN
Accuracy	95%	86%	89%
Precision	96%	84%	89%
Recall	94%	93%	93%
F1-Score	95%	89%	91%
AUC	99%	90%	95%

**Table 2:** Comparison of the three architectures in terms of the number of trainable parameters.

Architecture	Trainable Parameters
CNN-LSTM	456,865
MLP	22,526
KAN	127,206

## Data Availability

The data supporting the results reported in this study are available at https://github.com/michelalapenna/Convolutional-Network-models-for-supervised-quality-recognition-of-PPG-signals.git (accessed on 28 February 2025).
